# Physicochemical and Structural Properties of Gluten-Konjac glucomannan Conjugates Prepared by Maillard Reaction

**DOI:** 10.3390/polym15030631

**Published:** 2023-01-26

**Authors:** Yukang Song, Danping Huang, Wanchun Guo, Yiqing Gao, Feng Xue, Xiaohui Xiong, Chen Li

**Affiliations:** 1 College of Food Science and Light Industry, Nanjing Tech University, Nanjing 211816, China; 2 Nanjing Station of National Light Industry Food Quality Supervision and Inspection, Nanjing 211816, China; 3 School of Pharmacy, Nanjing University of Chinese Medicine, Nanjing 210023, China

**Keywords:** gluten, konjac glucomannan, Maillard reaction, physicochemical properties, structural properties

## Abstract

Gluten (Glu) is important to wheat products by forming a three-dimensional matrix. This study aimed to investigate the physicochemical and structural properties of gluten after conjugation with konjac glucomannan (KGM) through the Maillard reaction. The study revealed that the degree of graft increased with the prolonged reaction time. The Glu-KGM conjugates were possessed of increased β-sheet but decreased α-helix and β-turn, as well as unfolding and loose tertiary structures as the reaction proceeded. Among three different proportions, the Glu-KGM 1:1 conjugate was proved to have the most excellent foaming and emulsifying properties, and could form more rigid and firm gelation structures, which could be related to the decreased particle size and increased zeta potential of the conjugate. Overall, the physicochemical and structural properties of gluten were significantly related to the KGM ratios as well as the reaction period.

## 1. Introduction

Gluten is defined as the viscoelastic substance remaining in the wheat dough after starch granules are removed. It generates functional properties such as viscosity, elasticity, and water absorption to wheat products by forming a three-dimensional matrix [1]. According to the solubility of gluten in an aqueous solution, its components can be divided into glutenin and gliadin. The structure of glutenin can be divided into repeat region and non-repeat region. The repeat region is mainly formed by β-turn and exists in the form of α-helix, while the non-repeat region contains most cysteine residues and its main structure is α-helix. Gliadins are single histone proteins with an abundant network of cysteine residues held together by interchain disulfide bonds. The spiral structure is of great help to the formation of a viscoelastic network structure because of the high content of glutamine residues [2]. Gluten plays a very important role in bread making and is indispensable. The quantity and quality of protein have a great influence on the viscoelasticity of gluten, such as the volume, shape, and texture of protein [3], and balancing the extensibility and elasticity is the key to gluten network function [4].

In order to improve the quality of wheat dough, various water-soluble polysaccharides with different chemical structures and different functional characteristics such as gelation, thickening and water holding capacity can be used [5]. Konjac glucomannan (KGM) is a form of water-soluble colloid extracted from the tuber of Konjac. Its main components are D-mannose and D-glucose residues, and it is widely recognized due to its unique rheological and gelling properties as well as its many health benefits [6]. KGM has high water retention and good swelling, thickening, gelling, emulsifying, suspending, and cohesive properties, and its performance is superior to some gelling agents for food, such as agar, carrageenan, and gelatin. Therefore, it is often used in food processing, beverage preparation and cosmetics [7]. It is reported that adding KGM to protein such as minced fish, wheat and pork will change their physical and chemical properties and gelling properties. KGM, as a water-soluble hydrocolloid, can effectively promote the aggregation of protein by improving the hydrophobic interaction of protein, alleviate the thermal denaturation by reducing the flexibility of polypeptide chain, and alleviate the fluidity of gluten by stabilizing its structure [8]. In addition, in the gluten network structure, KGM can be uniformly dispersed to improve the stability and reduce the fluidity of dough, and therefore improve the texture of steamed bread [9]. 

The Maillard reaction, also known as glycation, is a non-enzymatic reaction which forms a covalent bond between protein and polysaccharide, and it has been demonstrated as an efficient method to improve protein functional properties [10]. The cross-linked product of protein and polysaccharide obtained by the Maillard reaction has obvious modified gelling properties [11,12]. At present, there is an increasing amount of research and discussion on KGM because of its influence on some physical and chemical properties of gluten. However, there is little data on the functional improvement of gluten through conjugation with KGM by the Maillard reaction, and the direct effects of KGM on the physicochemical properties of glycated gluten have been rarely studied at the molecular level.

The main components of dough are starch, gluten, and water, so it is a great challenge to study the interaction between gluten and KGM. Most of the experiments on Gluten-KGM (Glu-KGM) interactions have been performed in the dough, which contains both starch and gluten. This makes it difficult to determine interactions between dough ingredients or interactions involving KGM. In addition, it is necessary to focus on Glu-KGM interactions during thermal processing. To simplify matters, this work directly utilized gluten to determine the effect of KGM on its heat-induced changes. This study aimed to investigate the physicochemical and structural properties of Glu-KGM conjugates with different Glu/KGM ratios prepared through the Maillard reaction. The results can provide a theoretical basis for the application of Glu-KGM conjugates in wheat food.

## 2. Materials and Methods

### 2.1. Material 

Gluten from wheat (≥85%(protein) was purchased from Shanghai Yuanye Biotechnology Co., Ltd. (Shanghai, China). KGM (viscosity 15000 mpa.s) was supplied by Shanghai Aladdin Biochemical Co., Ltd. (Shanghai, China), which was used without further purification. O-phthalaldehyde, β-mercaptoethanol, and 8-aniline-1-naphthalenesulfonic acid (ANS) were purchased from Aladdin Biochemical Co., Ltd. (Shanghai, China). 2-Nitrobenzoic acid was purchased from Shanghai Macklin Biochemical Co., Ltd. (Shanghai, China).

### 2.2. Sample Preparation

Gluten protein and KGM were mixed thoroughly with a grinder according to the ratio of Glu:KGM as 1:1, 3:1, and 1:3, respectively. The mixtures were then placed in a desiccator filled with a saturated potassium bromide solution with a relative humidity of 79%, and kept at 65 °C for different times (0, 24, 48, and 72 h), then the mixtures were taken out and stored in a dryer. The samples with different proportions were recorded as Glu-KGM 3:1, Glu-KGM 1:1, and Glu-KGM 1:3, respectively. The gluten protein without any processing was used as control.

### 2.3. Degree of Graft (DG)

The DG value was analyzed according to the previous study with slight modifications [13]. OPA solution was prepared by adding 0.2 mL of β-mercaptoethanol, 50 mL of sodium tetraborate buffer (0.1 M, 250 mL, pH 9.18), and 5 mL 20% (*w*/*v*) sodium dodecyl sulfate (SDS) into 100 mL of distilled water. Then, 1% (*w*/*v*) of sample solution was added into 20 mL OPA solution, the solution was incubated for 10 min and measured for its absorbance at 340 nm. The DG value is calculated using Formula (1).

Degree of cross-linking (%) = (1 − (A_x_)/A_0_) × 100 (1)

where A_x_ is the content of free amino groups in the glycated samples and A_0_ is the content of free amino groups in the mixtures.

### 2.4. Color Measurement

The chromaticity of samples was measured using a spectrophotometer, L*, a* and b* values were recorded. Each sample was measured 5 times to obtain an average value [14]. The total color difference is represented by ∆E, which is calculated according to Formula (2).

(2) $$\Delta E=\sqrt{{\left(L^{*}-L\right)}^{2}+{\left(a^{*}-a\right)}^{2}{\left(b^{*}-b\right)}^{2}}$$

### 2.5. Particle Size and Turbidity Determination

The particle size was determined by dissolving a 2 mg/mL sample in 10 mL deionized water with a laser particle size analyzer (Malvern M3000, Malvern Instrument Ltd., Malvern, UK). The turbidity was determined by measuring the absorbance of sample dispersion (1 mg/mL) at 660 nm with a UV-visible spectrophotometry (UV-1900, Suzhou, China).

### 2.6. Secondary Structure 

The secondary structure of samples was analyzed using Fourier Transform Infrared Spectroscopy (Thermo Electron Corporation, Marietta, OH, USA) [15]. The samples were mixed with potassium bromide powder at 1:100 and then pressed into 1 mm tablets (10 mm diameter) in a stainless-steel cup. Infrared spectra (32 scans) were recorded for each prepared sample at room temperature with a resolution of 4 cm^−1^ in the range of 400–4000 cm^−1^. The software OMNIC spectrum analysis (Thermo Electron Corporation, Marietta, OH, USA) can effectively analyze and predict the content of secondary structure.

### 2.7. Measurement of Intrinsic Fluorescence Spectra

The samples were dissolved in phosphate buffer (0.01M, pH 7.2–7.4) to a final concentration of 0.15 mg/mL. Fluorescence measurements were performed on an F-4700 Fluorescence Spectrophotometer (Hitachi High-Tech Corporation, Toyko, Japan) equipped with FL Solutions software. 

### 2.8. Determination of Electric Potential

To determine the zeta potential, the sample was diluted to a concentration of 1 mg/mL, and then determined by Zeta potential analyzer (Malvern ZS90, Malvin Instrument Ltd., Malvern, UK).

### 2.9. Determination of Surface Hydrophobicity

Surface hydrophobicity was measured by the previously reported method [16]. The sample solution was diluted with phosphate buffer to five concentration gradients of 0.05, 0.1, 0.2, 0.5, and 1 mg/mL, respectively. Then, 50 μL of ANS was added to the solution, and the solution was incubated in a dark environment for 15 min. The fluorescence intensity of each sample solution was recorded at the excitation wavelength of 390 nm and the emission wavelength of 470 nm, respectively. The excitation and emission slit widths were both set to 5 nm. The fluorescence intensity was plotted against the sample concentration, and the initial slope of the curve was the surface hydrophobicity (H_0_) of the protein.

### 2.10. Foaming Capacity and Stability

Foaming stability and foamability were examined according to a previous study with minor modifications [17]. A total of 0.5 g of samples were dispersed into a beaker containing 50 mL of deionized water and were then homogenized with a disperser at 20,000 rpm for 2 min, then the solution was immediately poured into the measuring cylinder and its volume was record as V_1_. The total volume was recorded every 15 min within the range of 0 to 90 min. The final foaming capacity (FC) was calculated according to formula (3), and the foaming stability (FS) was calculated according to Formula (4).

FC = ((V_1_ − V_2_))/V_0_ × 100 (3)

where V_0_ is the total volume of the suspension, and V_2_ is the total volume of the suspension solution after 0 min of homogenization.

FS = V_t_/V_0_ × 100 (4)

where V_t_ is the foam volume at a certain time after homogenization.

### 2.11. Emulsifying Activity Index (EAI) and Emulsifying Stability Index (ESI)

The emulsifying properties of the samples were determined according to the method described by Yang et al. [13]. The oil-in-water emulsion was prepared using 4 mL of sample solution (2%, *w*/*v*) and 1 mL of vegetable oil and was then stirred for 2 min at 12,000 rpm by a disperser. 0.02 mL of the prepared emulsion was added to 5 mL of sodium dodecyl sulfate solution. Then, the absorbance of the mixed solution at 500 nm was read at 0 and 30 min, respectively. EAI is calculated by Formula (5) and ESI is calculated by Formula (6).

(5) $$\mathrm{EAI}\;(m^{2}/g)=\frac{\left(2\times \;T\;\times {\;A}_{0}\times \;\mathrm{dilution}\;\mathrm{factor}\right)\;}{\;\left(0.02\times \;\Phi \;\times 10^{4}\right)}$$

(6) $$\mathrm{ESI}\;(\min)=\frac{{A\;}_{0}}{\left[\left(A_{0}-{\;A}_{30}\right)\times 30\right]}$$

Here, T is 2.303, A_0_ and A_30_ are the absorbance values at 0 and 30 min, respectively, the dilution factor is 250, 0.02 is the initial concentration of protein (g/mL), and Φ is the volume of oil in the emulsion (0.2).

### 2.12. Rheological Properties

The rheological properties of the samples were determined by HR-1 rotational rheometer (TA Instruments, New Castle, PA, USA) with parallel plate geometry (60 mm diameter) and a 1mm gap, according to the reported method [9]. A frequency sweep was conducted from 0.1 to 16 Hz using constant strain (0.1 %, 25 °C), and the change curve of storage modulus (G′), loss modulus (G″), and loss factor tan δ (tan δ = G′/G″) was recorded.

### 2.13. Scanning Electron Microscopy (SEM)

The morphology of the samples was performed using a ZEISS Sigma 300 scanning electron microscope (Carl Zeiss AG, Oberkochen, Germany). The samples were sputter coated with gold-palladium and the images were obtained at a magnification of 10,000× and 3 KV accelerating voltage.

### 2.14. Statistical Analysis

All experiments were repeated at least three times, and the experimental results were obtained by a one-way ANOVA with the mean standard deviation using SPSS software (IBM, Armonk, NY, USA). All the figures were drawn by Origin 8.0 software (OriginLab, Northampton, MA, USA). Fisher’s least significant difference (LSD) test was used to calculate the difference between each pair of mean values, and the confidence level was 95% (*p*-value < 0.05).

## 3. Results and Discussions

### 3.1. DG Analysis

**Figure 1:**
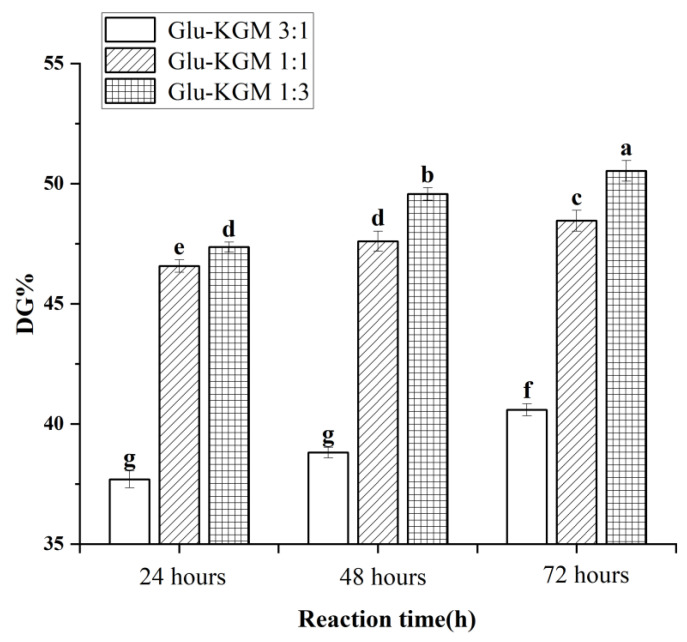
DG values of Glu-KGM conjugates with different proportions and reaction time. The results with different letters were significantly different (*p* < 0.05).

As free amino groups in protein molecules participated in the Maillard reaction, the decreasing degree of free amino groups could represent the DG value of the conjugates [18]. Figure 1 shows that the DG value of Glu-KGM conjugates gradually increased with the increase in reaction time. The DG value of the ratio of gluten to KGM of 3:1 was lower than that of 1:1 and 1:3, and the Glu-KGM 1:3 conjugates were possessed of the highest DG value at each reaction period. The results suggested that the higher the KGM content, the higher the glycation degree between gluten and KGM.

### 3.2. Color Change

**Figure 2:**
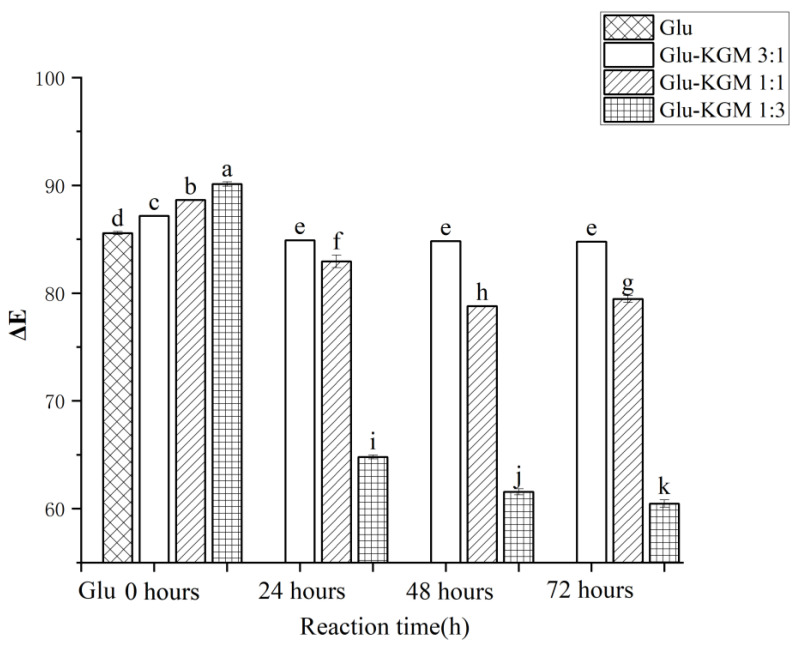
Total color changes of gluten, mixtures, and conjugates, respectively. The results with different letters were significantly different (*p* < 0.05).

The extent of the Maillard reaction can be judged by the color change. As shown in Figure 2, ΔE of Glu-KGM mixtures with different proportions decreased significantly after 24 h of reaction, especially when the ratio of gluten to KGM was 1:3. The main reason may be that more KGM may result in a more obvious Maillard reaction with gluten. After 48 h of reaction, it can be seen that the ΔE of the Glu-KGM 1:1 and Glu-KGM 1:3 conjugates continued to decrease, while the ΔE of Glu-KGM 3:1 conjugate stayed unchanged compared with that of 24 h reaction, which may be due to the Maillard reactions having been completed. After 72 h of the reaction, it can be seen that the ΔE of the Glu-KGM 1:1 and Glu-KGM 1:3 conjugates slightly changed. By comparing the ΔE among the conjugates, it can be seen that the higher the KGM content, the darker the color, and the faster the glycation rate of Glu-KGM conjugates.

### 3.3. Particle Size and Turbidity

**Figure 3:**
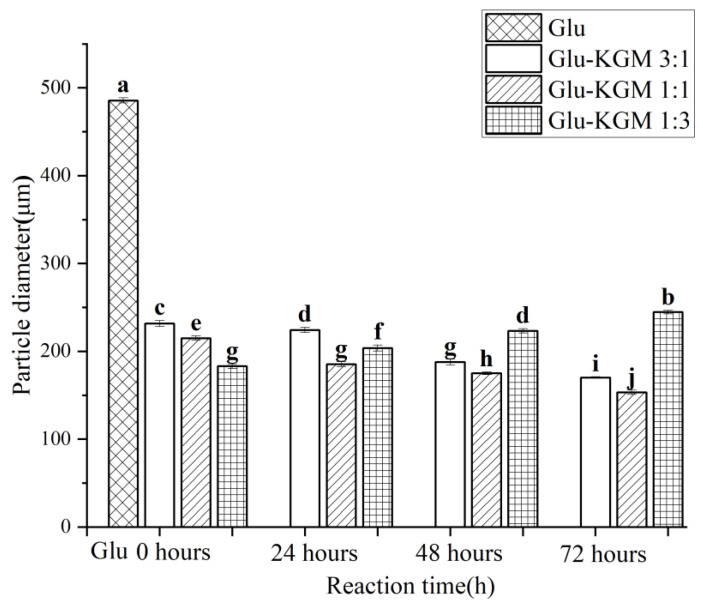
Particle size distribution diagram of gluten, mixtures, and conjugates, respectively. The results with different letters were significantly different (*p* < 0.05).

**Figure 4:**
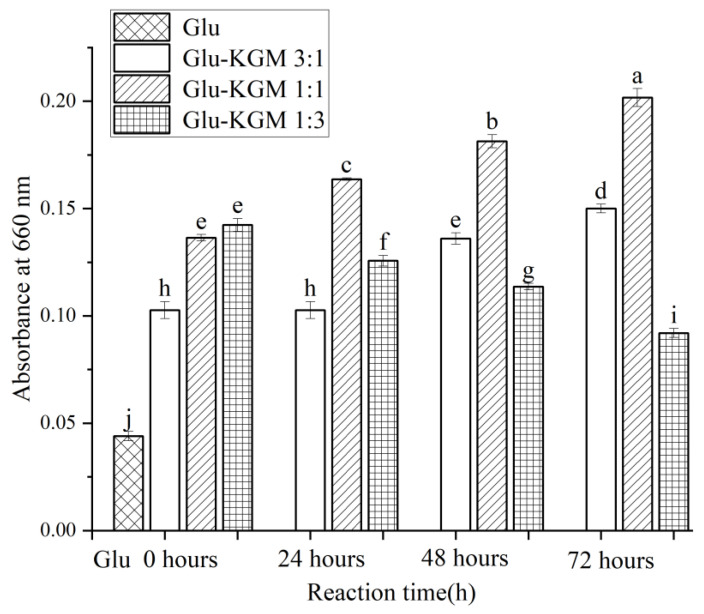
Turbidity diagram of gluten, mixtures, and conjugates, respectively. The results with different letters were significantly different (*p* < 0.05).

Figure 3 clearly shows that the size of gluten is much larger than that of gluten added with KGM. This indicates that the addition of KGM polysaccharide can effectively inhibit the swelling of gluten and reduce its particle size significantly, which may be due to the glycation reaction between gluten and KGM, which caused the hydrogen bond between gluten and KGM to regenerate. The hydrogen bond shifts and redistributes, thus changing the hydrophobic interaction of gluten. Because of its low solubility in water, gluten is very prone to agglomeration. After the glycation reaction, KGM could effectively inhibit the swelling of gluten in water and could enhance the anti-deformation ability of gluten, thereby reducing its particle size [19]. The changes in particle size were opposite to the changes in turbidity (Figure 4). This may be because the smaller the particle size, the greater the degree of obstruction of the solution to the passage of light, therefore more light scattering through the conjugate being formed, leading to higher turbidity of the cross-linked gluten conjugates [16].

### 3.4. Secondary Structure

The secondary structure is one of the essential conformational indicators of gluten. The bands at 1600–1644^−1^ and 1685–1700 cm^−1^ are attributed to β-sheets, while 1644–1652 cm^−1^ are random coils. The regions located at 1652–1660 cm^−1^ and 1660–1685 cm^−1^ are α-helix and β-turn, respectively [15]. In gluten, β-sheets and β-turns have the highest relative numbers, followed by α-helices and random coils. However, for gliadin and glutenin, α-helix is usually located at the end of N- and C-, and β-turn and intermolecular β-fold exist in the helix’s central repeat domain. [20].

**Figure 5:**
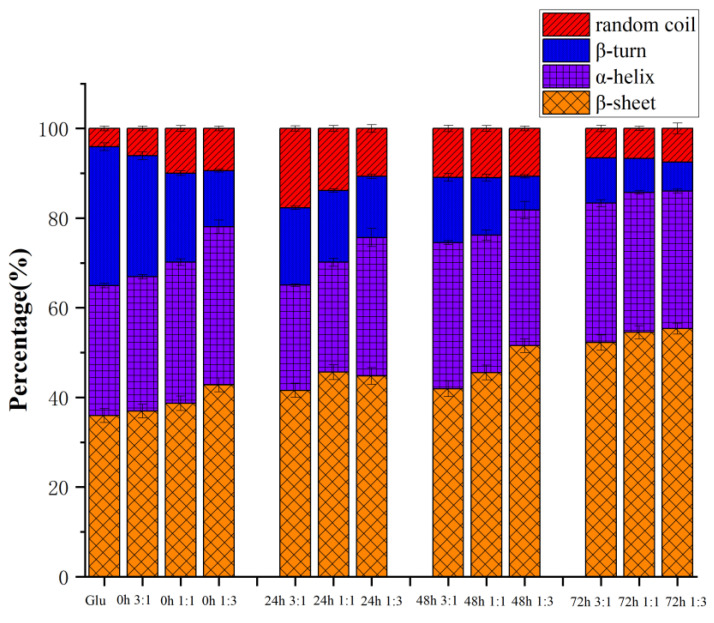
The secondary structure of gluten, mixtures, and conjugates, respectively. The results with different letters were significantly different (*p* < 0.05).

As shown in Figure 5, the content of the β-sheet of gluten before heating was higher than that of β-turn and α-helix, and after conjugating with KGM, it can be seen that the content of the β-sheet significantly increased. This may be enhanced by breaking the strong hydrogen bonds of the tight α-helix. When gluten is heated, the secondary structure of gluten is mainly determined by the number of hydrogen bonds, hydrophobic interactions, and disulfide bonds [21]. The hydrogen bond can be expanded by heat treatment, and reassembled by hydrophobic interaction and disulfide bond, thus the secondary structure can be rearranged [22]. After 72 h of the reaction, the proportion of β-sheet significantly increased, while the proportion of β-turn significantly decreased. The above results showed that during the heating process, the cross-linking of gluten and KGM hindered the unfolding process of disulfide bonds, thereby reducing the breakage of disulfide bonds, and causing the molecular chains to polymerize to form macromolecular cross-links. When the heating time was limited (24 h), KGM did not significantly change the secondary structure of gluten, and it is possible that a long heating time can fully aggregate, fold, and arrange the molecular chains [23]. It has been reported that β-sheet and β-turn contents were positively correlated with gluten viscoelasticity, while α-helix contents were negatively correlated. From this, it is inferred that KGM could promote gluten aggregation and dough rheological properties through the Maillard reaction. The glycation of KGM could reduce the α-helix/β-sheet content ratio of gluten and make the gluten structure more stable [24].

### 3.5. Intrinsic Fluorescence Intensity

The emission spectrum can provide information about the microenvironment of fluorescent amino acids, and it can be regarded as a sensitive indicator to characterize protein through its conformation, kinetics, and intermolecular interaction. The measurements of endogenous fluorescence spectroscopy are mainly used to detect conformational changes in proteins around Trp residues. To further confirm the conformational change of gluten during glycation, its intrinsic fluorescence spectrum was obtained at an excitation wavelength of 280 nm [25].

**Figure 6:**
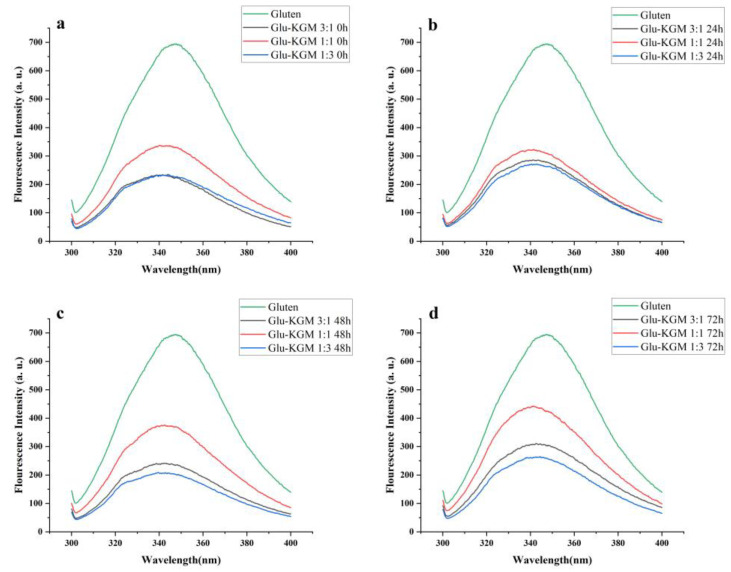
Intrinsic fluorescence spectra of gluten, mixtures, and conjugates, respectively. (**a**) Glu-KGM 3:1, Glu-KGM 1:1, and Glu-KGM 1:3 mixtures; (**b**) Glu-KGM 3:1, Glu-KGM 1:1, and Glu-KGM 1:3 conjugates heated for 24h; (**c**) Glu-KGM 3:1, Glu-KGM 1:1, and Glu-KGM 1:3 conjugates heated for 48 h; (**d**) Glu-KGM 3:1, Glu-KGM 1:1, and Glu-KGM 1:3 conjugates heated for 72 h.

Figure 6 shows that, compared with gluten, the intrinsic fluorescence intensity of Glu-KGM mixtures and conjugates was significantly lower than that of gluten, indicating that KGM had a certain shielding effect on the region around Trp residues. The fluorescence spectrum of both Glu-KGM mixtures and conjugates showed a blue-shift of λ_max_ compared to that of gluten. The reason may be due to the attachment of KGM to the interior of gluten, which inhibited the exposure of Trp residues to solvent. The result is consistent with the result of surface hydrophobicity (Section 3.7) [26]. Moreover, the fluorescence spectrum of Glu-KGM conjugates after 72 h glycation all showed a red-shift of λ_max_ compared to that of the mixtures. The λ_max_ of Glu-KGM mixtures with the ratio of 3:1, 1:1, and 1:3 was 341.4, 341.4, and 344 nm, respectively, and the λ_max_ of Glu-KGM conjugates after 72 h reaction with the ratio of 3:1, 1:1, and 1:3 was 342.6, 341.6, and 344.6 nm, respectively. The red-shift of the wavelength indicated that the spatial structure around Trp became looser. Therefore, the gluten structure could unfold during the heating process, resulting in the exposure of hidden tryptophan residues.

### 3.6. Zeta Potential

**Figure 7:**
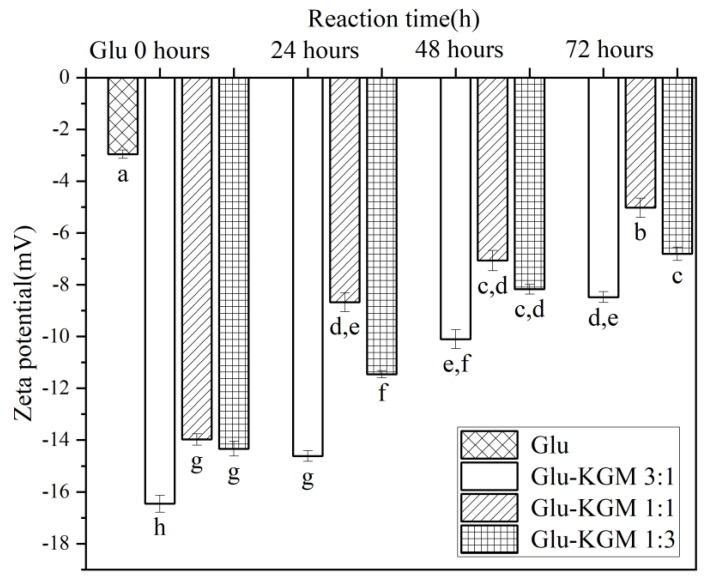
Zeta potential of gluten, mixtures, and conjugates, respectively. The results with different letters were significantly different (*p* < 0.05).

Zeta potential is an index reflecting the surface charge of protein molecules in a water environment, which can characterize the conformational change of protein. [13]. As can be seen in Figure 7, the zeta potential of gluten decreased significantly after adding KGM, indicating that the structure of wheat gluten became unstable after adding KGM. Another possible reason for the decrease is that protein molecules and KGM aggregate in the process of glycation and cross-linking, so that negatively charged amino acid residues are buried. After the glycation treatment, it was found that the zeta potential increased with the increase in heating time. In the process of glycation, the combination of KGM and gluten may lead to an electrostatic shielding effect and the change of the surface charge of gluten, thus reducing the zeta potential [27].

### 3.7. Surface Hydrophobicity(H_0_)

**Figure 8:**
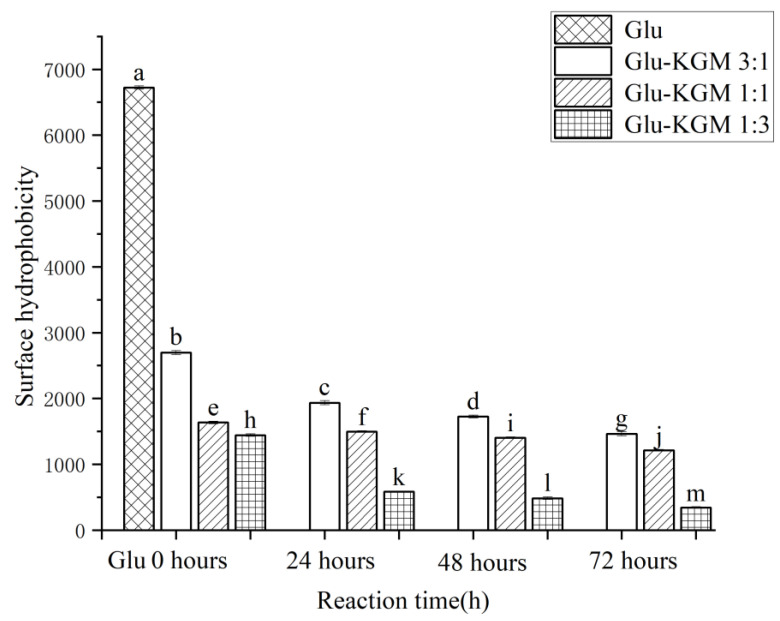
Surface hydrophobicity of gluten, mixtures, and conjugates, respectively. The results with different letters were significantly different (*p* < 0.05).

Through the specific binding with ANS, the degree of hydrophobic groups on gluten surface can be determined and expressed as H_0_ value. The surface hydrophobicity would decrease if the gluten unfolds due to heating and then aggregates through hydrophobic interactions, thereby reducing the number of ANS binding sites [21]. As shown in Figure 8, it can be seen that the surface hydrophobicity of wheat gluten decreased significantly after adding KGM, while the surface hydrophobicity of Glu-KGM conjugates gradually decreased with the increase in heating time. The higher the KGM content, the lower the hydrophobicity. This decrease in surface hydrophobicity indicated that KGM polysaccharide exhibited a significant shielding effect after the glycation reaction, which prevented ANS from binding to hydrophobic groups inside gluten. Proteins undergo irreversible intermolecular aggregation through hydrophobic interactions and disulfide bonds, resulting in protein folding that masks the hydrophobic groups, which in turn triggers a drop in H_0_ value during heating [28]. In some cases of heating, the hydrophobic groups on the surface of the unfolded protein can be exposed by heating, thus increasing H_0_. Therefore, the unfolding and intermolecular aggregation can occur simultaneously in the heating process, thus affecting the H_0_ value [29].

### 3.8. Foaming Capacity and Stability

Foaming performance includes foam capacity (FC) and foam stability (FS). It can play the role of aeration and whipping in the food production process. The interfacial film formed by whipping protein, and its ability to keep bubbles suspended and slow down coalescence, is the key to foam formation [30]. Figure S1 shows the foaming properties of Glu-KGM mixtures and conjugates. It can be seen that the foaming ability of Glu-KGM mixtures is better than that of wheat gluten. Moreover, compared with the Glu-KGM 3:1 conjugate, the Glu-KGM 1:1 and Glu-KGM 1:3 conjugates had higher foaming stability. This is because that higher KGM content resulted in increasing the surface viscosity and surface strength of the liquid film [17]. The foaming ability of Glu-KGM conjugates increased with the increase in glycation reaction time, and the foaming capacity of the Glu-KGM 1:1 conjugate was the highest. This may be related to the change in particle size, that a protein with smaller particles could absorb faster in the process of stirring to produce foam. With the decrease in protein particles, the elasticity of the interfacial film formed by protein can be effectively increased, resulting in more foam [14]. The foaming ability of Glu-KGM conjugates was consistent with the changing trend of particle size. Therefore, it can be seen that the addition of KGM can significantly improve the foaming stability and foaming ability of gluten.

### 3.9. EAI and ESI

**Figure 9:**
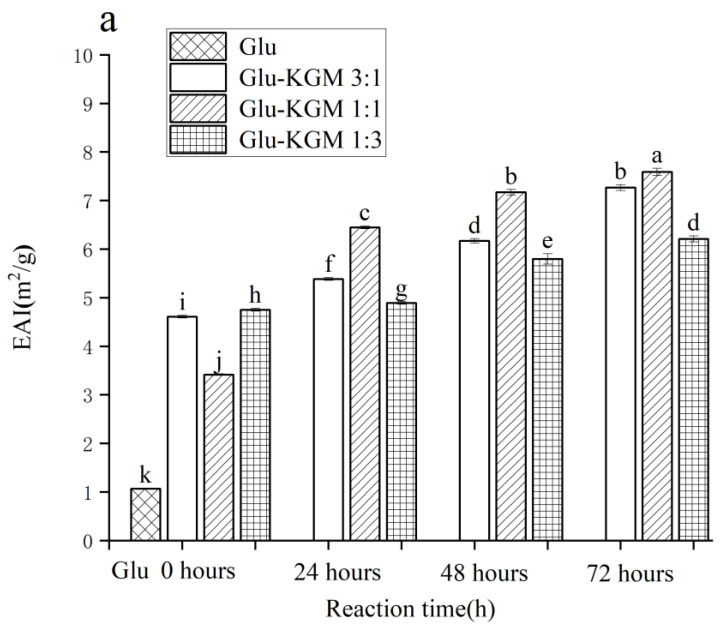

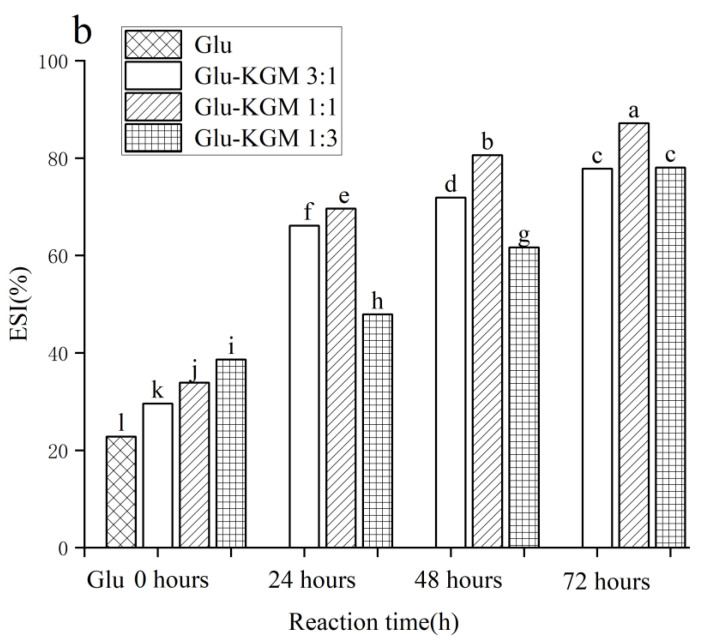
Emulsifying activity and emulsifying stability of gluten, mixtures, and conjugates, respectively. (**a**) Emulsifying activity of gluten, mixtures, and conjugates, respectively. (**b**) Emulsifying stability of gluten, mixtures, and conjugates, respectively. The results with different letters were significantly different (*p* < 0.05).

In the process of emulsion formation, EAI shows the adsorption capacity of proteins at the oil-water interface [31]. In Figure 9a, it can be seen that the EAI value of gluten is 1.065 m^2^/g. After adding KGM, EAI was significantly improved, and it increased with the increase in reaction time. After 72 h of reaction, the EAI value of the Glu-KGM 1:1 conjugate was the highest. During the Maillard reaction, the structure of the protein could swell and become more flexible, which can increase its emulsifying activity. In addition, the average particle size in the Glu-KGM 1:1 conjugate was the smallest, which can form a thicker interfacial layer to improve the stability of steric orientation [32].

The ability of protein to maintain the stability of emulsion and inhibit the coalescence of oil droplets can be reflected by ESI. Factors that affect EAI and ESI are different. EAI is mainly related to surface hydrophobicity, whereas ESI usually depends on the surface charge of proteins [33]. As proteins can covalently cross-link with polysaccharides, their functional characteristics at the oil-water interface can be improved. Protein molecules can quickly adsorb to the oil-water interface to form an amphiphilic film, while long-chain polysaccharides can play a protective role, increasing the thickness of the film around the oil droplets, thus reducing the polymerization and flocculation of protein molecules and improving the performance stability of the emulsion [34]. As shown in Figure 9b, the ESI of gluten was significantly lower than that of the Glu-KGM mixtures and conjugates. Therefore, the Glu-KGM conjugates could form an interface film and promote the dispersion of oil droplets, which was the result of the unfolding of the conjugates structures [35]. The ESI values of the Glu-KGM conjugates increased with the increase in reaction time. The results indicated that the addition of KGM made the interfacial film around the oil droplets more stable and less prone to coalescence, which may be due to the further glycation enhancing the hydrophobicity of the conjugates, thereby improving the stability of the oil/water interface [36,37]. Among them, the ESI value of the Glu-KGM 1:1 conjugate was the highest, which was 87.17% after 72 h of reaction, followed by 80.63% after 48 h of reaction.

### 3.10. Rheological Properties

As shown in Figure S2, ′the G′ and G″ values of all samples increased with the frequency in the range of 0.1 to 16 Hz. The value of G″ is lower than that of G′, which indicates that gluten, Glu-KGM mixtures, and conjugates have viscoelastic and solid-like properties [9]. The G′ value and G″ value of Glu-KGM mixtures were significantly lower than those of the control group, indicating that KGM had a significant negative effect on the dynamic modulus of gluten. The result was consistent with previous reports [38]. The reason for this may be that the addition of KGM disturbed the continuous gluten network structure, in which noncovalent interactions, such as the hydrogen bond, was destroyed, resulting in the decrease in gluten strength during dough formation [39]. After glycating with KGM, the G′ and G″ value of the Glu-KGM conjugates further decreased. This may be due to the decrease in charge density (Figure 7), which reduced the electrostatic repulsion [40]. However, the G′ and G″ of the Glu-KGM 1:1 conjugate after 24 h and 48 h reaction both increased as compared to the Glu-KGM 1:1 mixture. The results showed that the covalent bonding of polysaccharides could expose more functional groups and, at the same time, it could reduce the particle size of cross-linked products, which made gluten protein cross-linking more easy [41]. In addition, the increase in G′ values with increasing frequency confirmed the Glu-KGM 1:1 conjugate, after 24 h and 48 h of reaction, possessed the firm texture of gelation.

Tan δ is the ratio of G″ to G′, which can reflect the quality of protein, and its value can reflect the deterioration degree of protein. The larger the value, the higher the deterioration degree of protein. The range of tanδ value was 0.1 to 1.2 (Figure S2g–h), indicating that gluten was solid in the test frequency range, while KGM with high viscosity can make the network structure of gluten more elastic and rigid [25]. The above results showed that with the increase in KGM concentration, the stability viscosity of the Glu-KGM conjugates’ micelles increased. The tan δ of the Glu-KGM 3:1 conjugate was positively correlated, which indicated that it cannot significantly improve the stability of the gluten structure, thus effectively reducing the viscosity and fluidity of gluten. However, the tan δ of the Glu-KGM 1:1 and Glu-KGM 1:3 conjugates were negatively correlated, which indicated that the stability of the gluten structure was improved, and the viscosity and fluidity of the gluten decreased. Among them, the Glu-KGM 1:1 conjugate after 48 h of the reaction possessed the lowest tan δ value, presenting the best stability. The reason may be that the network structure of the Glu-KGM 1:1 conjugate after 48 h of the reaction was more elastic and rigid, thus hindering the deterioration of gluten [42].

### 3.11. Microscopic Analysis

Figure S3a shows a highly porous structure of gluten. Figure S3b–d shows that after adding KGM, the surface of the Glu-KGM mixtures became rougher, and formed the aggregation of lumpy particles. With the increase in heating time and the increase in KGM content, the porous network structure became blurred, and a layered structure was observed after glycation. This may be because KGM was attached to the surface of the gluten system, weakening the network structure of gluten. These findings suggested the formation of a strong Glu-KGM network after glycation, and gluten fraction may contribute more to the shape and strength of the network [43].

## 4. Conclusions

Physicochemical and structural properties of Glu-KGM conjugates for different proportions and different reaction durations prepared from the Maillard reaction were investigated. The results suggested that cross-linking of KGM could greatly inhibit the expansion and rupture of gluten particles while leading to relatively loose spatial structures. The KGM could help in forming the Glu-KGM network structure after glycation. Moreover, the ratio of gluten to KGM should be appropriate, in order to obtain improved functional properties of the conjugates. The Glu-KGM 1:1 conjugate was demonstrated to have the most excellent foaming properties, emulsifying properties, and more rigid and firm gelation structures, which was significantly correlated with the decreased particle size and increased zeta potential of the conjugate. The above results provided useful references for the future application of Glu-KGM conjugates in wheat-related food. Future research should focus on applying Glu-KGM conjugates in frozen dough or bakery products.

## Data Availability

The data presented in this study are available on request from the corresponding author.

## Supplementary Materials

The following supporting information can be downloaded at: https://www.mdpi.com/article/10.3390/polym15030631/s1, Figure S1: Foaming properties of gluten, mixtures and conjugates, respectively; Figure S2: Rheological properties of gluten, mixtures and conjugates, respectively; Figure S3: Electron micrograph of gluten, mixtures and conjugates, respectively.
