# Supplementary material for: Physicochemical and Structural Properties of Gluten-Konjac glucomannan Conjugates Prepared by Maillard Reaction
Source: Polymers (Basel). 2023 Jan 26;15(3):631. doi: 10.3390/polym15030631 (PMC9921320; doi:10.3390/polym15030631)
Supplement: Supplementary file 1 [file polymers-15-00631-s001.zip › polymers-2106745-supplementary.pdf]

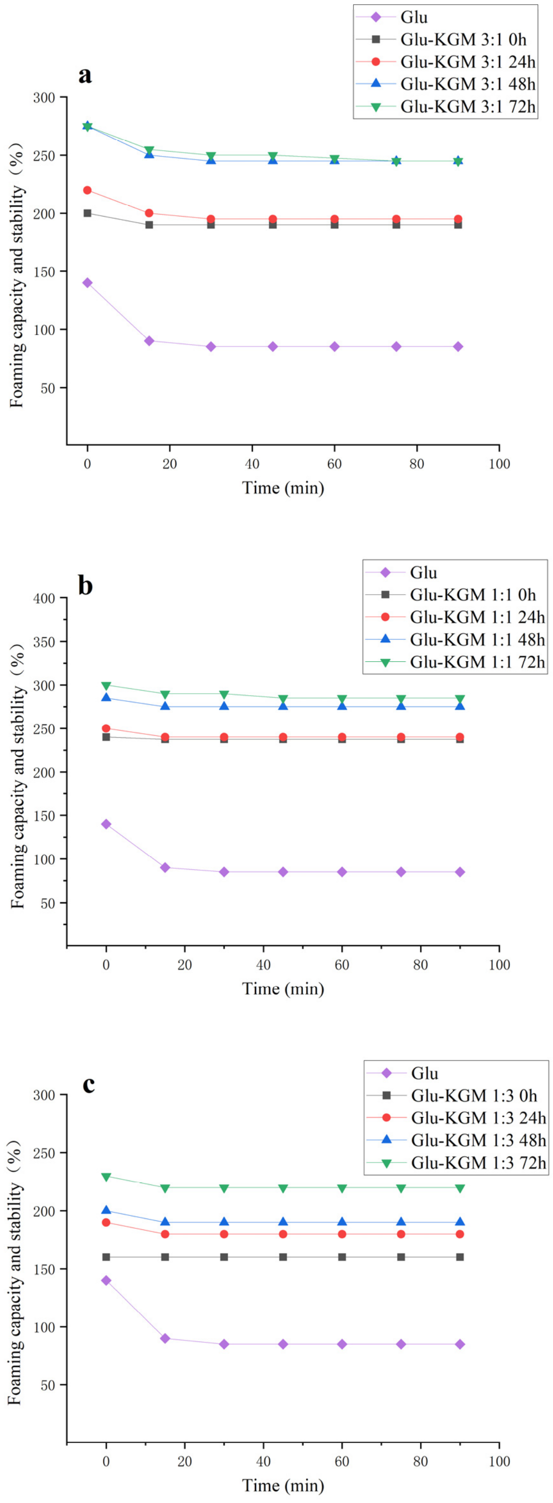

Figure S1. Foaming properties of gluten, mixtures and conjugates, respectively. a: Gluten, Glu-KGM 3:1 mixtures and conjugates; b: Gluten, Glu-KGM 1:1 mixtures and conjugates; c: Gluten, Glu-KGM 1:3 mixtures and conjugates.

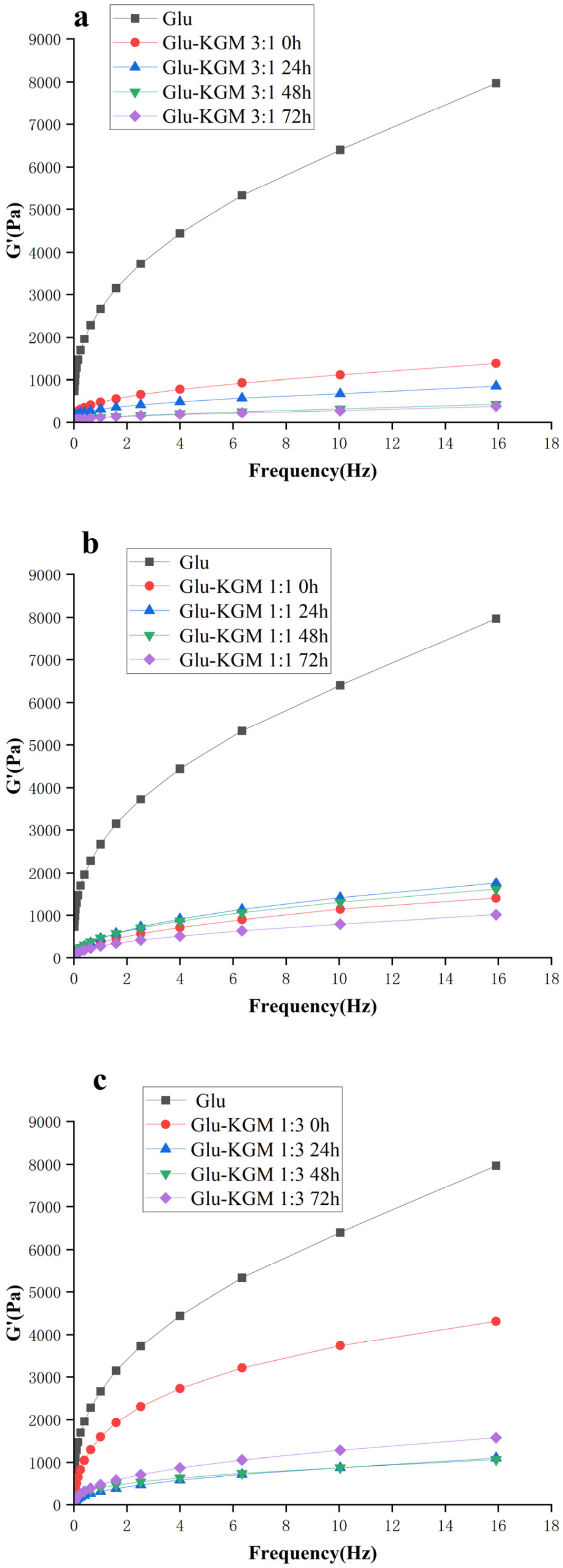
a
Glu
Glu-KGM 3:1 0h
Glu-KGM 3:1 24h
Glu-KGM 3:1 48h
Glu-KGM 3:1 72h
G'(Pa)
Frequency(Hz)
b
Glu
Glu-KGM 1:1 0h
Glu-KGM 1:1 24h
Glu-KGM 1:1 48h
Glu-KGM 1:1 72h
G'(Pa)
Frequency(Hz)
c
Glu
Glu-KGM 1:3 0h
Glu-KGM 1:3 24h
Glu-KGM 1:3 48h
Glu-KGM 1:3 72h
G'(Pa)
Frequency(Hz)

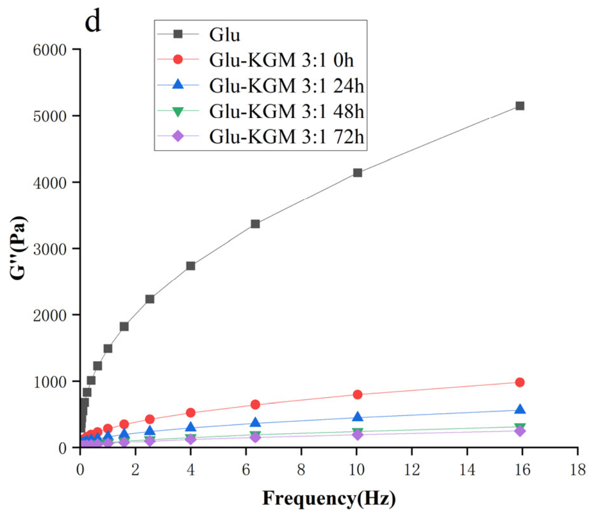

d
Glu
Glu-KGM 3:1 0h
Glu-KGM 3:1 24h
Glu-KGM 3:1 48h
Glu-KGM 3:1 72h
G''(Pa)
Frequency(Hz)

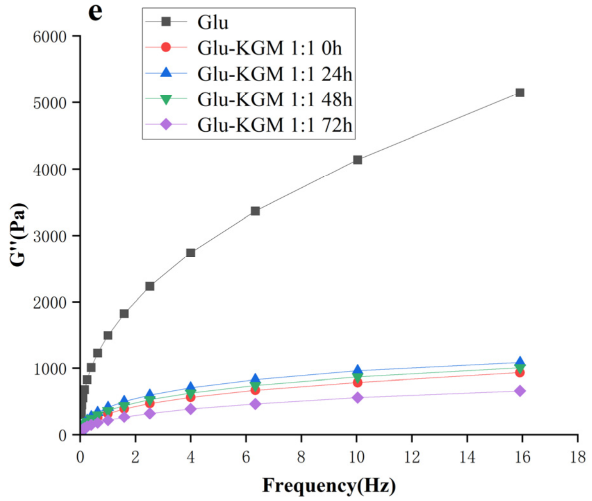

e
Glu
Glu-KGM 1:1 0h
Glu-KGM 1:1 24h
Glu-KGM 1:1 48h
Glu-KGM 1:1 72h
G''(Pa)
Frequency(Hz)

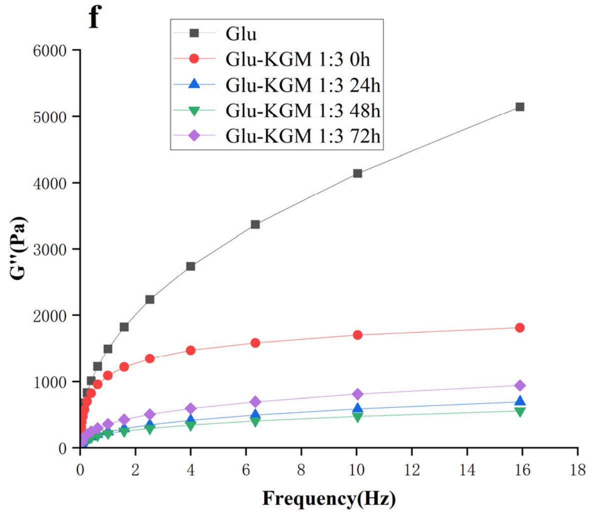

f
Glu
Glu-KGM 1:3 0h
Glu-KGM 1:3 24h
Glu-KGM 1:3 48h
Glu-KGM 1:3 72h
G''(Pa)
Frequency(Hz)

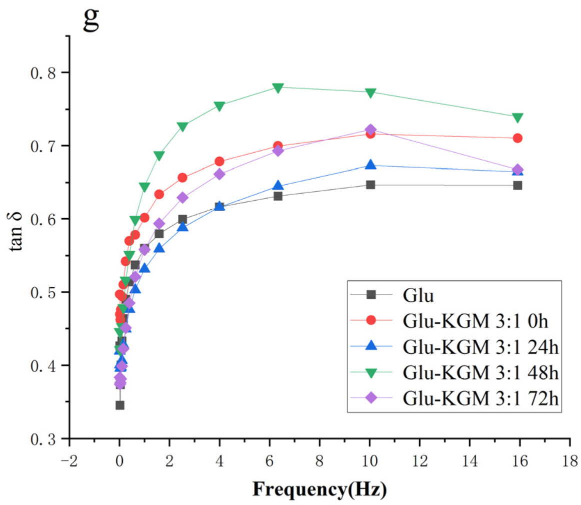
g
tan δ
Frequency(Hz)
Glu
Glu-KGM 3:1 0h
Glu-KGM 3:1 24h
Glu-KGM 3:1 48h
Glu-KGM 3:1 72h

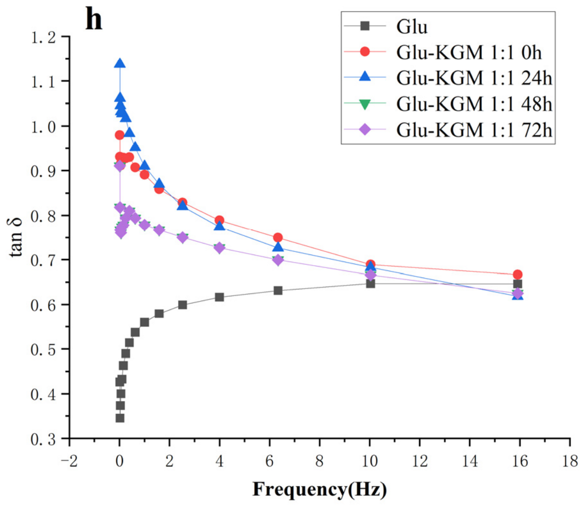
h
tan δ
Frequency(Hz)
Glu
Glu-KGM 1:1 0h
Glu-KGM 1:1 24h
Glu-KGM 1:1 48h
Glu-KGM 1:1 72h

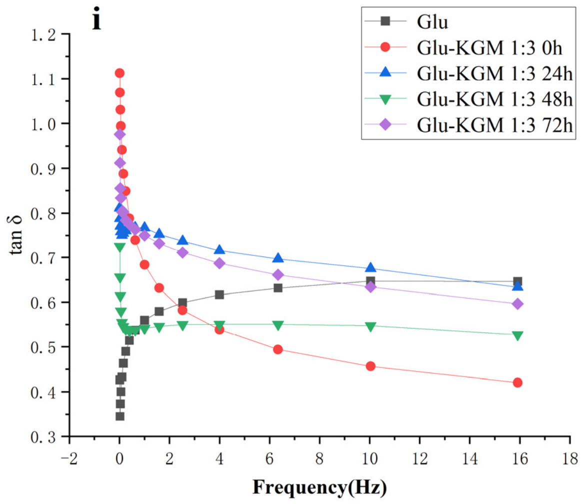
i
tan δ
Frequency(Hz)
Glu
Glu-KGM 1:3 0h
Glu-KGM 1:3 24h
Glu-KGM 1:3 48h
Glu-KGM 1:3 72h

Figure. S2. Rheological properties of gluten, mixtures and conjugates, respectively. G′, elastic modulus (Figure a-c), G'', viscous modulus (Figure d-f), and tan δ, loss tangent (Figure g-i).

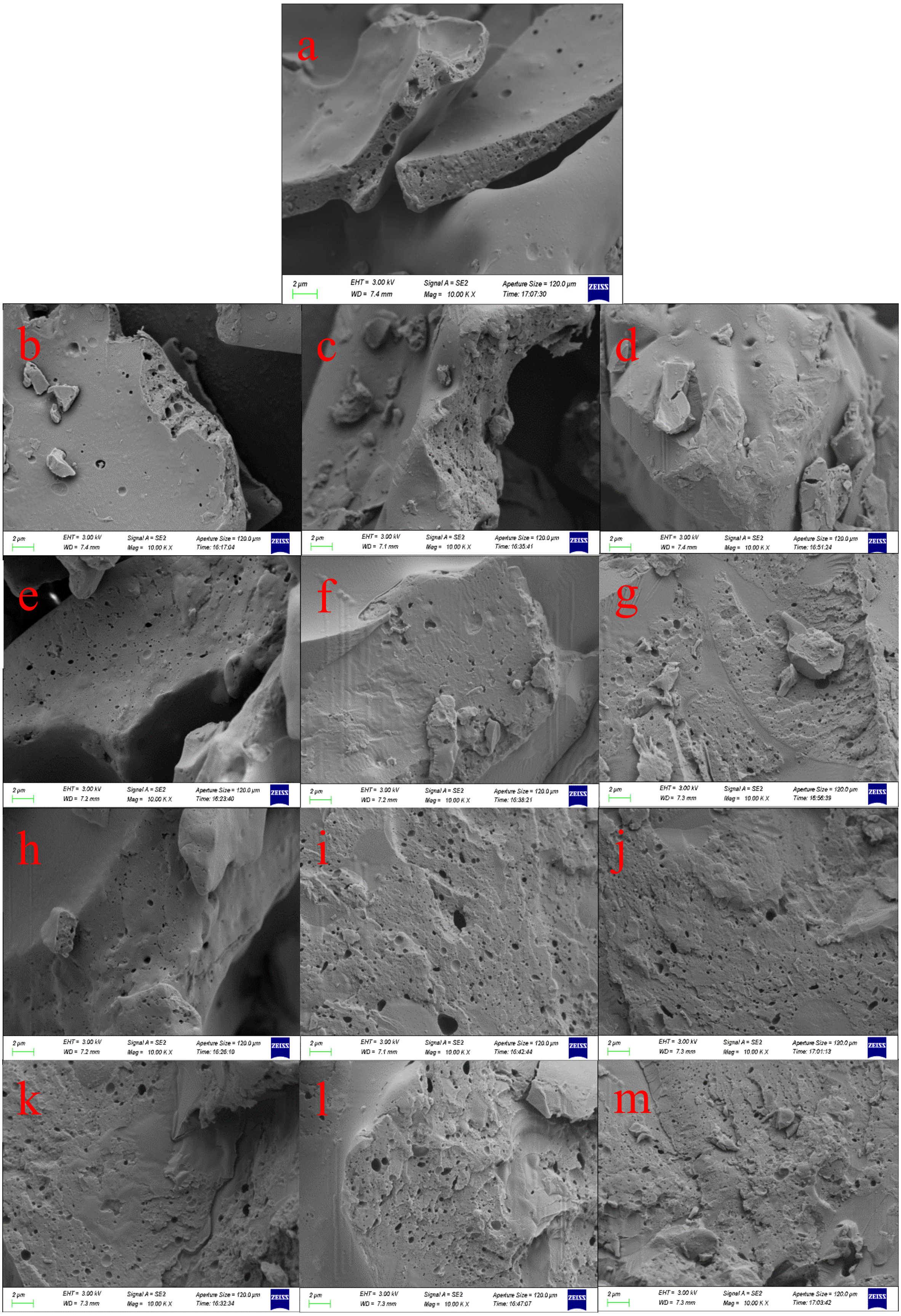

Figure S3. Electron micrograph of gluten, mixtures and conjugates, respectively. a: Gluten; b-d: Glu-KGM 3:1, Glu-KGM 1:1, and Glu-KGM 1:3 mixtures; e-g: Glu-KGM 3:1, Glu-KGM 1:1, and Glu-KGM 1:3 conjugates heated for 24h; h-j: Glu-KGM 3:1, Glu-KGM 1:1, and Glu-KGM 1:3 conjugates heated for 48h; k-m: Glu-KGM 3:1, Glu-KGM 1:1, and Glu-KGM 1:3 conjugates heated for 72h.
